# Thrombopoietin as Biomarker and Mediator of Cardiovascular Damage in Critical Diseases

**DOI:** 10.1155/2012/390892

**Published:** 2012-04-05

**Authors:** Enrico Lupia, Alberto Goffi, Ornella Bosco, Giuseppe Montrucchio

**Affiliations:** ^1^Department of Clinical Pathophysiology, University of Turin, via Genova 3, 10126 Turin, Italy; ^2^Critical Care Department, Saint Michael's Hospital, Toronto, ON, Canada M5B 1W8

## Abstract

Thrombopoietin (TPO) is a humoral growth factor originally identified for its ability to stimulate the proliferation and differentiation of megakaryocytes. In addition to its actions on thrombopoiesis, TPO directly modulates the homeostatic potential of mature platelets by influencing their response to several stimuli. In particular, TPO does not induce platelet aggregation *per se* but is able to enhance platelet aggregation in response to different agonists (“priming effect”). Our research group was actively involved, in the last years, in characterizing the effects of TPO in several human critical diseases. In particular, we found that TPO enhances platelet activation and monocyte-platelet interaction in patients with unstable angina, chronic cigarette smokers, and patients with burn injury and burn injury complicated with sepsis. Moreover, we showed that TPO negatively modulates myocardial contractility by stimulating its receptor c-Mpl on cardiomyocytes and the subsequent production of NO, and it mediates the cardiodepressant activity exerted *in vitro* by serum of septic shock patients by cooperating with TNF-**α** and IL-1**β**. This paper will summarize the most recent results obtained by our research group on the pathogenic role of elevated TPO levels in these diseases and discuss them together with other recently published important studies on this topic.

## 1. Introduction

Thrombopoietin (TPO) is a humoral growth factor originally identified using antisense oligonucleotides to c-mpl [1], a protooncogene that is the human homolog of *v-mpl*, the viral oncogene responsible for the transforming myeloproliferative leukemia virus (MPLV) [2]. Initial characterization of c-Mpl-deficient mice showed that they have severe thrombocytopenia but relatively normal levels of other hematological cell types [3]. Subsequently, several research groups identified TPO as the primary ligand for the c-Mpl receptor [4–10] and TPO/Mpl signal transduction was shown to play critical roles in thrombopoiesis, from *ex vivo* megakaryocyte progenitor expansion and differentiation to *in vivo* platelet production [4–11].

TPO is constitutively produced in the liver and kidneys and is then cleared from circulation upon binding with its receptor c-Mpl [7, 8]. Upon TPO binding, c-Mpl receptors undergo homodimerization to initiate intracellular signaling, including activation of the JAK2/signal transducers and activators of transcription (STAT) pathway [12].

In addition to its role in thrombopoiesis, TPO also plays a role in expanding erythroid and granulocytic-monocytic progenitors [13], and loss of TPO/Mpl signaling was associated to a potential defect in the multipotent cell compartment or even in the stem cell compartment [14, 15]. Subsequent studies clearly defined the role of TPO in expanding or maintaining the pool of transplantable hematopoietic stem cells, further establishing the responsiveness of cells in the primitive hematopoietic compartment to this cytokine signaling pathway [16, 17].

Elevated plasma TPO levels have been reported in different clinical conditions, including several hematological diseases usually associated with thrombocytopenia, where increased circulating TPO may be a response to altered bone marrow hematopoiesis or bone marrow failure [18–20]. Of particular importance is the example of immune thrombocytopenia, a disorder characterized by immune-mediated platelet destruction and impaired platelet production, resulting in platelet count lower than 100,000 per cubic millimeter and varying degrees of bleeding risk [21]. In patients with immune thrombocytopenia, indeed, TPO levels are usually normal or only slightly increased for reasons that remain unclear [19, 22]. This observation led to the concept of treating the disorder by means of exogenous stimulation of TPO receptors and to the development of TPO-receptor agonists, whose clinical use was recently approved for adult patients affected by immune thrombocytopenia at risk for bleeding [21, 23].

Besides hematological diseases, elevated circulating TPO levels have been also reported in other clinical conditions, including critical diseases such as acute coronary syndromes [24, 25] and sepsis [26–29] (Table 1). This paper will summarize the most recent results obtained by our research group on the pathogenic role of elevated TPO levels in these diseases and discuss them together with other recently published important studies on this topic.

## 2. Effects of Thrombopoietin on Platelet Activation

Shortly after the cloning and characterization of TPO as c-Mpl ligand, we and others have shown that the TPO receptors are expressed by mature platelets and that TPO directly modulates the homeostatic potential of platelets by influencing their response to several stimuli [30, 31]. In particular TPO does not induce platelet aggregation* per se*, both in platelet-rich plasma and in whole blood, but is able to enhance platelet aggregation in response to different agonists (“priming effect”) [30, 31]. In addition, TPO stimulates platelet-leukocyte associations in whole blood through expression of platelet P-selectin [32]. The characterization of this “priming effect” prompted us to study the pathophysiological effects of plasma TPO in those clinical conditions where TPO levels are increased and platelet activation has a pathogenic role.

## 3. Thrombopoietin Increases Platelet Activation in Patients with Unstable Angina

It is known that patients with unstable angina (UA) have hypersensitive platelets, increased levels of circulating platelet aggregates and platelet secretory products [33–37]; they also show increased platelet-leukocyte aggregates, which have been proposed to represent a better reflection of plaque instability and ongoing vascular thrombosis and inflammation [38, 39]. Activated platelets deposit indeed at sites of unstable plaque rupture and may potentiate thrombus formation, precipitating or exacerbating coronary vascular obstruction [35]. The clinical efficacy of antiplatelet therapies also confirms the importance of platelets in acute coronary syndromes [40]; however, their incomplete effectiveness suggests that alternative platelet activation pathways may be important [40].

Higher levels of TPO were previously reported by Senaran and colleagues [24] in patients with acute coronary syndromes than control subjects and shown to correlate with platelet size, thus potentially resulting in hemostatically more active platelets [24].

In our study [25], we enrolled 15 patients with unstable angina (UA) and, as controls, 15 patients with stable angina (SA) and 15 healthy subjects. We measured circulating levels of TPO by ELISA and of C-reactive protein (CRP) by immunoturbidimetric assay, as well as *ex vivo *monocyte-platelet binding and the platelet expression of P-selectin and of the TPO receptor, c-Mpl, by flow cytometry [41]. Finally, the priming activity of patient or control plasma on platelet aggregation and monocyte-platelet binding [25] and the role of TPO in this effect were also studied *in vitro*.

Confirming what was previously reported by others [24], we found higher circulating TPO levels in patients with UA, at the time of hospital admission, than in patients with SA or healthy controls [25]. Of note, we enrolled the UA patients in the emergency room, before any therapeutic intervention was started, and we therefore believe that our data may closely reflect the ongoing pathogenic events leading to the development of UA.

Elevation of TPO in the plasma of patients with UA was also indirectly confirmed by the finding of reduced surface expression of c-Mpl on circulating platelets, whereas no such a change was detected in patients with SA or healthy controls [25]. Receptor-mediated internalization is indeed considered the primary mean of regulating plasma TPO levels [42, 43]. Diminished c-Mpl expression in platelets from UA patients may thus depend on the previous binding of TPO to its receptor *in vivo*, followed by its internalization and surface downregulation [42, 43]. In addition, the *in vitro *stimulation with recombinant human TPO (rhTPO) was able to phosphorylate c-Mpl only in platelets from SA patients and healthy controls, but not in those from UA patients, further sustaining this hypothesis [25]. This same mechanism was previously shown for thrombocytopenic mice [44] and humans with the syndrome of congenital thrombocytopenia with absent radii [45], whose platelets are exposed, *in vivo* to increased levels of endogenous TPO.

Decreased surface levels of c-Mpl together with decreased platelet sensitivity to exogenous stimulation *in vitro *with a pegylated N-terminal domain of human TPO (PEG-rHuMGDF) have also been evoked as a “protective” mechanism against the prothrombotic effects of *in vivo* treatment with PEG-rHuMGDF in a rat model of mesenteric microthrombosis [46]; however, the conclusions of this study refer only to thrombocytopenic states, in which endogenous TPO levels are elevated [44–46], whereas this and our subsequent studies were addressed to specific pathologic conditions in which TPO levels rise in presence of normal platelet counts. The precise origin of the rise in plasma TPO level in UA patients remains unclear. We found that CRP levels were increased in this study group, suggesting that the liver acute-phase response, which takes place in acute coronary syndromes [47–49], may have a role in increasing TPO levels. Considering the evidence that elevated CRP has independent prognostic value in UA [50, 51], it is tempting to speculate that the negative prognostic implications of high CRP levels in patients with UA may be at least partially related to the concomitant increase in TPO production and subsequent priming of platelet aggregation. However, activated platelets could also represent a major contributor to the elevated TPO levels observed in UA patients, since they are known to release full-length biological active TPO upon stimulation [52]. In addition, platelet alpha-granular proteins may increase TPO gene expression and consequent TPO production in bone marrow stromal cells via a feed-back mechanism [53, 54].

In addition to elevated plasmatic concentrations of TPO, patients with UA also showed increased indexes of *ex vivo* platelet activation, such as monocyte-platelet binding and platelet P-selectin expression [25].

The presence of TPO in the circulation precludes the evaluation of its role on platelet aggregation directly on blood samples obtained from patients. Therefore, we studied the contribution of TPO to platelet aggregation by adding patient plasma samples to platelets of healthy subjects *in vitro* and inhibiting TPO biological activity by using a TPOR-Fc chimera synthesized in our laboratory [25]. In these experimental conditions, plasma from patients with UA, but not from SA patients or healthy controls, markedly enhances platelet aggregation as well as monocyte-platelet binding in blood samples from healthy donors, stressing the importance of elevated TPO concentrations in the pathogenesis of increased platelet aggregation in UA [25]. Several data show that the “priming effect” exerted by plasma samples from patients with UA may be due to their content in TPO: (1) the priming effect induced by plasma from UA patients was significantly decreased when TPO activity was inhibited with the TPOR-Fc chimera; and (2) adjusting the concentrations of TPO in plasma from SA patients to those measured in UA patients by adding exogenous rhTPO induced a significant increase of the priming effect, similar to that observed with plasma from patients with UA, on ADP- or EPI-induced aggregation in PRP. *In vivo*, a similar “priming effect” induced by TPO on platelet activation has also been documented in nonhuman primates; platelets derived from TPO-treated animals showed indeed a heightened sensitivity to substances that stimulate platelet aggregation during the first few days of treatment [55].

Plasma from UA patients also induced a significant priming effect on platelet aggregation in the presence of acetylsalicylic acid or in PRP from healthy subjects after one-week oral acetylsalicylic acid treatment [25]. These results suggest that the activation pathway triggered by TPO is only partially affected by the antiplatelet therapy commonly used in patients with myocardial ischemia [40] and that platelet priming by TPO may represent a mechanism leading to therapeutic failure of antiplatelet agents. Moreover, the phenomenon we described in this study may provide the rationale for more aggressive (double or triple) antiplatelet treatment in patients with acute coronary syndromes.

In conclusion, in this study we showed that elevated levels of circulating TPO may enhance platelet activation and monocyte-platelet interaction in the early phases of UA [25]. Our *ex vivo* and *in vitro* findings suggest an important link between circulating TPO level and the pro-inflammatory and prothrombotic state that occurs in UA patients and implicate TPO in the pathogenesis of acute coronary syndromes, where it could potentially precipitate conditions of clinical instability.

## 4. Thrombopoietin Increases Platelet Activation in Cigarette Smokers

Chronic smoking is a major risk factor for the development of atherosclerosis and thrombosis, and it has been strongly associated with adverse cardiovascular effects [56]. In particular, enhanced platelet aggregability and subsequent alterations in the clotting cascade have been evoked [57, 58] as main pathogenic factors sustaining the increased risk of coronary artery thrombosis in long-term smokers [57, 59]. Since inflammation plays a central role in the pathogenesis of atherosclerosis and its complications [60], a number of studies have investigated the association between smoking and increase in several inflammatory markers, such as CRP [61], interleukin (IL)-6 [62], tumor necrosis factor (TNF)-*α* [62], and CD40L [63]. However, the biological mechanisms linking smoking and atherosclerosis are complex and have not been fully elucidated.

In our study [64], we evaluated TPO and CRP levels, platelet-leukocyte binding and the platelet expression of P-selectin, and the priming activity of smoker or control plasma on *in vitro* platelet-monocyte binding in 20 healthy cigarette smokers and 20 age- and gender-matched nonsmokers. In a second phase of the study we investigated the effects of acute smoking and of smoking cessation on TPO levels and *ex vivo* platelet activation markers [64]. For this purpose, healthy non-smoking subjects were studied at the baseline and after they smoked two cigarettes in 30 minutes. Moreover, 8 healthy cigarette smokers were studied at the baseline and after they had quit smoking for three weeks.

In the first part of this study, we found that chronic smokers have higher circulating TPO levels than nonsmokers and higher platelet-leukocyte binding and platelet P-selectin expression *ex vivo* [64]. Although cigarette smokers had significantly higher TPO levels than nonsmokers, the concentrations measured in both groups were lower than those measured in patients with UA [25] and, may someway be considered within the “physiological” range (i.e., in our experience, below 40 pg/mL).

In addition to elevated plasmatic concentrations of TPO, cigarette smokers also showed increased indices of *ex vivo* platelet activation, such as higher platelet-monocyte binding and platelet P-selectin expression [64]. Moreover, TPO levels correlated with *ex vivo* platelet-monocyte aggregation and P-selectin expression [64].

*In vitro*, plasma from cigarette smokers, but not from nonsmoking subjects, significantly enhanced platelet-monocyte binding in blood samples from healthy donors [64]. The contribution of TPO to this priming effect is suggested by the correlation analysis showing that TPO levels and platelet-monocyte adhesion in whole blood consensually increased in the two groups. Moreover, the direct proof that TPO participates to the platelet activation observed in chronic smokers is provided by the inhibitory effect of the TPOR-Fc chimera [64]. Taken together, our data support the hypothesis that circulating TPO may facilitate platelet activation in smokers by sensitizing platelets to the action of other agonists. Therefore, it can be suggested that TPO is required, but is not sufficient *per se* to promote aggregate formation, and cooperates with other mediators to induce the observed changes on platelet activation in chronic smokers. Interestingly, TPO primes platelet-monocyte binding in chronic smokers even in the presence of increased levels of CRP, which is known to inhibit platelet aggregation and platelet capture of leukocytes [65]. On the contrary, plasma samples from cigarette smokers did not induce a significant priming effect on platelet aggregation both in PRP and whole blood. This discrepancy with the results obtained using flow cytometry may depend on the different sensitivity of these techniques.

Since smoking may also “acutely” affect platelet function [66], we evaluated whether “acute” smoking (two cigarettes in the previous 30 minutes) was able to induce an increase in TPO levels and platelet activation. We found that the changes in TPO levels upon “acute” smoking, in our experimental conditions, were about a half of the increase observed in chronic smokers compared with non-smoking controls [64]. However, this TPO elevation did not affect *ex vivo* platelet activation, evaluated in terms of platelet-leukocyte adhesion and P-selectin expression [64]. These results suggest that transient platelet activation and consequent TPO release by activated platelets induced by “acute” smoking have only a negligible role in causing the increase in platelet activation markers observed in long-term smokers. In addition, “acute” smoking plasma failed to promote EPI-induced platelet-leukocyte binding and P-selectin expression in *in vitro *experiments [64]. This result can be due to several reasons: (a) the short exposure of the subjects to cigarette smoking (all of them were not usual smokers), (b) the relatively low amounts of TPO released after “acute” smoking, which are further diluted in the *in vitro* experiments, and also (c) the lack of the concomitant presence of other factors which can be released after chronic exposure to smoking and cooperate with TPO in enhancing platelet activation.

Alternatively, higher TPO levels in chronic smokers may depend on increased hepatic synthesis, sustained by the liver acute-phase response that takes place in inflammatory diseases, including atherosclerosis [60]. It is known that IL-6, the main acute-phase reactant produced in the liver, enhances TPO synthesis [67]. The findings that chronic smokers have elevated CRP concentrations and that CRP increases concomitantly with TPO would be in agreement with this hypothesis. However, in our study, we were not able to document a concomitant decrease in TPO and CRP concentrations after smoking cessation [64], and we have no direct data sustaining the hepatic origin of TPO in our subjects. Therefore, the precise origin of the rise in plasma TPO level observed in cigarette smokers remains unclear.

Further stressing the importance of chronic exposure to smoking products in the genesis of platelet function abnormalities, chronic cigarette smokers who quit smoking for three weeks showed a significant decrease in TPO levels, which was associated with reduced platelet-monocyte and platelet-granulocyte bindings [64]. Consistently, plasma drawn after smoking cessation induced a significantly lower platelet-monocyte aggregation *in vitro *compared to that induced by plasma obtained before smoking cessation [64]. On the contrary, no differences in platelet-granulocyte adhesion and P-selectin expression were observed [64].

In conclusion, the results of this study suggest that TPO may contribute to enhance platelet activation and platelet-monocyte cross-talk in cigarette smokers and that increased TPO may represent a novel pathogenic mechanism whereby cigarette smoking promotes atherogenesis and is associated with the development of adverse cardiovascular events [64].

## 5. Thrombopoietin Increases Platelet Activation in Patients with Burn Injury and Burn Injury Complicated with Sepsis

Several investigations show that dysregulation of the TPO/Mpl receptor system is also present in sepsis. In particular, elevated TPO levels have been reported in healthy volunteers after endotoxin infusion [28], as well as in septic children and neonates [26, 68–71] and septic adult patients [27, 29]. More recently, Zakynthinos and colleagues showed that TPO levels are greatly increased in patients with sepsis compared to control subjects and correlate with sepsis severity and that sepsis severity represents the major determinant of elevated TPO levels in these patients [29]. To investigate the potential contribution of elevated TPO levels in platelet activation during burn injury complicated or not by sepsis, we studied 22 burned patients, 10 without and 12 with sepsis, and 10 healthy subjects [72]. We measured plasma levels of TPO, as well as leukocyte-platelet binding and P-selectin expression *in vivo*, and assessed the “priming” activity of plasma from burned patients or healthy subjects on platelet aggregation and leukocyte-platelet binding and the involvement of TPO in these effects* in vitro* [72].

In this study we found that burn injury is associated with a significant increase in the circulating levels of TPO, about twofold the levels measured in healthy subjects [72]. TPO levels further increase upon development of sepsis, suggesting that the development of sepsis, in addition to burn injury, may contribute to increase circulating TPO levels in these patients [72]. These results are substantially in agreement with those already reported by Zakynthinos and colleagues in a larger population of patients with sepsis [29].

We also found that patients with burn injury show increased monocyte-platelet aggregates and platelet P-selectin expression, compared to healthy subjects [72]. In addition, monocyte-platelet aggregates were significantly higher in burned patients with sepsis than burned patients without sepsis [72]. These findings showed that increased platelet activation (i.e., P-selectin expression) and heterotypic aggregation (i.e., monocyte-platelet adhesion) also occur in burn injury, especially after sepsis development, suggesting that activated platelets amplifies the inflammatory reactions and favor the insurgence of organ damage in these pathological conditions.

The precise origin of the rise in TPO levels observed in burned patients without and with sepsis remains unclear. TPO levels are well known to be primarily regulated by platelet mass [73–75], and yet we did not detect thrombocytopenia in septic patients in our study. Burned patients without and with sepsis also showed increased indices of *in vivo* platelet activation compared to healthy subjects. Moreover, we found a positive correlation between (a) TPO levels and monocyte-platelet binding* in vivo* and (b) TPO levels and platelet P-selectin expression *in vivo*. Therefore, platelets themselves may represent a major contributor to increased TPO levels, or high TPO levels in burned and septic patients may depend on increased hepatic synthesis.

Analogously to previous studies, we studied the contribution of TPO to platelet aggregation by adding patient plasma samples to platelets of healthy subjects *in vitro* and inhibiting TPO biological activity by using the TPOR-Fc chimera [72]. In these experimental conditions, plasma from burned patients without and with sepsis, but not from healthy subjects, enhances platelet aggregation as well as monocyte-platelet binding and platelet P-selectin expression in blood samples from healthy donors [72]. The “priming effect” induced by plasma from burned patients with sepsis was significantly higher than that induced by plasma from burned patients without sepsis in all the experimental conditions tested [72]. The contribution of TPO to the “priming effect” exerted by plasma samples from burned patients without and with sepsis is suggested by (1) the correlation analysis showing that TPO levels and ADP- and EPI-induced priming index in PRP and whole blood consensually increased in the three groups; (2) the inhibitory effect of the TPOR-Fc chimera [72]. Taken together, our *in vivo* and *in vitro *data support the hypothesis that TPO present in the circulation of burned patients, especially those developing sepsis, may facilitate platelet activation by sensitizing circulating platelets to the action of other agonists, thus precipitating the occurrence of microvascular thrombosis and the clinical onset of multiorgan failure.

In conclusion, increased TPO levels may enhance platelet activation during burn injury and sepsis and have a role in the pathogenesis of multiorgan failure in these pathological conditions.

## 6. Effects of Thrombopoietin on Myocardial Cell Contractility and Implications for Cardiac Dysfunction in Patients with Septic Shock

Myocardial dysfunction is common in patients with sepsis and is associated with high risk to develop multiorgan failure and high mortality rate [76–78]. Septic cardiomyopathy is characterized by reversible biventricular dilatation, decreased ejection fraction, and impaired response to fluid resuscitation and catecholamine stimulation [76–78]. Although also intrinsic cardiac factors have been implicated in this complex condition [76–78], the causal role of circulating factors has been extensively studied [77–79], following the observation that serum from patients with septic shock decreases myocyte contractile function and that this effect correlates with the reduction of the patient's left ventricular ejection fraction [80]. A pivotal role for TNF-*α* and IL-1*β* in mediating this depressant activity has clearly emerged from subsequent studies [81]. Each individual cytokine, although at supraphysiological concentrations, as well as the combination of the two at concentrations similar to those measured in the bloodstream of septic patients, is able to reproduce *in vitro* the depressant effect of septic serum [82]. This response is mainly mediated by the production of nitric oxide (NO) and cyclic guanosine monophosphate (cGMP) [83], although also NO-independent mechanisms have been involved [84].

No data on the potential contribution of TPO to myocardial depression during sepsis was available at the time. We therefore planned an experimental *in vitro *study to investigate whether TPO affects myocardial contractile function and contributes to the myocardial depressing activity of septic shock serum [85].

A first original result of this study was the demonstration of the presence of the TPO-receptor c-Mpl in the rat heart by RT-PCR and immunoblotting and in human myocardium by immunoblotting [85]. Since endothelial cells express c-Mpl [7], we studied the presence of c-Mpl also in cultured cardiomyocytes, definitely showing that they express c-Mpl on the cellular surface, as also confirmed by confocal microscopy [85].

We then evaluated the effects of TPO on myocardial contractility *in vitro* and found that TPO did not directly modify the contractile force of isolated rat papillary muscle, but blunted the enhancement of contractile force induced by epinephrine (EPI) in both papillary muscle and isolated heart preparations [85]. We previously observed similar TPO effects in other experimental settings, where TPO exerts no direct action *per se*, but rather amplifies the effects of other biological mediators, in particular when we studied platelet aggregation and monocyte-platelet interaction in response to ADP and EPI [30], or the production of oxygen free radicals by polymorphonuclear leukocytes challenged with fMLP [86].

The specificity of TPO effect was assessed by inhibiting the biological activity of TPO using a TPOR-Fc chimera, which completely abrogated the effects exerted by TPO on EPI-induced cardiac contractility, whereas it did neither alter the contractile force of papillary muscle or isolated heart nor influence their responsiveness to beta-adrenergic stimulation [85].

The occurrence of myocardial depression in septic shock is a well-documented phenomenon, which is associated with high mortality rate [76–78]. Interestingly, the major pathogenic mechanisms of septic cardiac dysfunction include both alterations of adrenergic response and the effects of humoral mediators [76–79]. Myocardial hyporesponsiveness to catecholamines (including decreased chronotropy and inotropy) has been shown in several endotoxic models of septic shock [87, 88], as well as in human septic shock [89, 90]. This effect has been related to disruption of *β*-adrenergic signal transduction in cardiomyocytes due to both NO-dependent and -independent mechanisms [84], analogously to what was reported for ischemia/reperfusion injury [91–93]. Our results suggest that also TPO may influence the contractile response elicited by EPI stimulation by affecting adrenergic signal transduction [85]. Moreover, the results obtained by using specific pharmacological inhibitors on isolated rat papillary muscles show that TPO action is mediated by the activation of the PI3K-Akt1-NO Synthase-Guanylyl Cyclase pathway, which leads to the production of NO as final mediator [85]. We have also shown that TPO directly induces the phosphorylation of Akt1 in H9C2 cardiomyocytes and isolated papillary muscles [85].

Evidence of a circulating myocardial depressant substance in the serum of septic shock patients was first demonstrated by Parrillo et al. [80]. Subsequent studies indicated TNF-*α* and IL-1*β* as the pivotal mediators inducing cardiac depression in sepsis [82] and other disease conditions [94, 95]. On the basis of the recent reports of elevated TPO levels in sepsis [26–29, 68–72], we hypothesized that TPO may concur to depress myocardial contractility during sepsis. We observed that pretreatment of serum samples with the TPOR-Fc chimera completely prevented the decrease in contractile force-induced by human sepsis shock serum alone, whereas it had no effect on serum of healthy subjects [85]. In addition, we found that the negative inotropic effect of both TNF-*α* and IL-1*β* was significantly enhanced by the addition of TPO [85]. Moreover, TPOR-Fc chimera completely prevented the decrease in contractile force induced by addition of TPO to TNF-*α* or IL-1*β*, whereas it did not modify the contractile responses induced by these cytokines alone [85]. The results obtained strongly suggest that high levels of circulating TPO in patients with sepsis may favor the occurrence of myocardial depression in cooperation with TNF-*α* and IL-1*β*.

In conclusion, we showed in this study that TPO negatively modulates myocardial contractility by stimulating its receptor c-Mpl on cardiomyocytes and the subsequent production of NO [85]. In addition, TPO mediates the cardiodepressant activity exerted *in vitro* by serum of septic shock patients, which is indeed completely abrogated by the TPOR-Fc chimera [85]. Finally, TPO cooperates with TNF-*α* and IL-1*β* in depressing cardiac contractility [85]. Therefore, our results suggest that TPO may have a relevant role in modulating cardiac inotropy in septic shock by affecting the two major pathogenic mechanisms described: (a) by influencing adrenergic receptor signal transduction; (b) by cooperating with circulating mediators known to reduce myocardial contractility, namely TNF-*α* and IL-1*β*.

## 7. Characterization of Thrombopoietin as a Physiological Regulator of Coronary Flow

We have previously shown that human umbilical cord vein-derived endothelial cells (HUVECs) expressed the TPO receptor c-Mpl and that TPO activates HUVECs *in vitro*, as indicated by directional migration, synthesis of platelet-activating factor and IL-8, and phosphorylation of STAT1 and STAT5B [96]. Others have shown that specific murine liver EC (LEC-1) located in the hepatic sinusoids coexpresses TPO and its receptor, c-Mp, and that TPO has a proliferative effect on LEC-1 [97]. Furthermore, stimulation with TPO induced secretion of proinflammatory cytokines (i.e., IL-1*β*, IL-6, TNF-*α*) from LEC-1, some of which cooperate with TPO in sustaining the proliferation of these cells [98].

Based on these data, we sought to investigate the potential role of TPO in coronary flow modulation and to determine the mechanisms involved.

The expression of TPO receptor c-Mpl and the TPO-dependent eNOS phosphorylation (P^Ser1179^) were showed on cardiac-derived normal human microvascular endothelial cells (HMVEC-C) by Western blot analysis [99]. While TPO (10–200 pg/mL) did not modify coronary flow (CF) under basal conditions, it reduced the coronary constriction caused by endothelin-1 (ET-1; 10 nM) in a dose-dependent manner [99]. This effect was blocked by both Wortmannin (100 nM) and L-NAME (100 nM); on HMVEC-C, TPO induced eNOS phosphorylation through a Wortmannin sensitive mechanism [99]. Our data suggest a potential role of TPO as a physiological regulator of CF. By acting on specific receptors present on endothelial cells, TPO may induce PI3K/Akt-dependent eNOS phosphorylation and NO release.

## 8. Effects of Thrombopoietin Treatment on Myocardial Cell Viability and Cardiac Function in Experimental Models of Myocardial Ischaemia-Reperfusion Injury and Heart Failure

Recent experimental studies by other groups reported results apparently not consistent with ours [100–102].

Li and colleagues [100], based on the rationale that TPO possesses antiapoptotic functions mediated by the Akt prosurvival axis in hematopoietic stem cells and megakaryocytes [103, 104], hypothesized that TPO may protect against cardiotoxicity induced by doxorubicin. In their study, they showed that TPO exerts antiapoptotic activity in two different *in vitro* cellular models, namely, the fetal rat cardiomyocyte cell line H9C2 and spontaneously beating primary neonatal rat cardiomyocytes [100]. Moreover, TPO was able to preserve cardiac functions, including heart rate, fractional shortening, and cardiac output, evaluated by echocardiography, in an *in vivo *model of doxorubicin-induced acute cardiotoxicity [100].

More recently, the same research group extended their previous results by studying cardiac damage in two different rat models of acute- and chronic-doxorubicin treatment [102]. In both models TPO treatment led to significant improvements of fractional shortening, cardiac output, and morphologic parameters [102]. In the acute-doxorubicin model, microarray and network analyses showed that cardiac damage was associated with changes in a large cohort of gene expressions, of which many were inversely regulated by TPO, including modulators of signal transduction, ion transport, antiapoptosis, protein kinase B/p42/p44 extracellular signal-regulated kinase (AKT/ERK) pathways, cell division, and contractile protein/matrix remodeling [102]. Many of these regulations also occurred in the animals chronically treated with doxorubicin, in which TPO treatment reduced morphological damage and cardiomyopathy score, and increased AKT phosphorylation of heart tissues [102]. TPO was also shown to increase the formation of endothelial progenitor cell (EPC) colonies in their bone marrow [102]. The conclusions of the authors suggest that TPO-induced cardiac protection from acute- and chronic-doxorubicin damages may be mediated by multifactorial mechanisms including AKT- and ERK-associated restoration of regulatory gene activities critical for normal heart function [102].

In another recent study, Baker and colleagues demonstrated that TPO treatment immediately before ischaemia reduced myocardial necrosis, apoptosis, and decline in ventricular function following ischaemia/reperfusion both *in vitro *in the rat isolated heart and *in vivo *[101]. This TPO effect is concentration and dose dependent with an optimal concentration of 1.0 ng/mL *in vitro* and an optimal dose of 0.05 *μ*g/kg iv *in vivo* [101]. Increased resistance to injury from myocardial ischaemia/reperfusion conferred by TPO has been shown to be mediated by JAK-2, p42/44 MAPK, and K_ATP_ channels [101].

## 9. Discussion and Conclusions

We have no clear explanation for the discrepancy between the results summarised in the last paragraph [100–102] and ours [85], although the different experimental models, together with the concentrations used, well above the physiological range, may, at least partially, justify these results.

 In our study [85] we evaluated indeed TPO cardiac effects at doses analogous to those measured in human pathology, in particular during septic shock [29], whereas, for instance, Li and colleagues used TPO concentrations as high as 50 ng/mL or 100 ng/mL [100], which are commonly reported as effective in promoting hematopoietic cells in culture [105, 106]. Moreover, in our study [85] we focused on the acute changes induced by TPO pretreatment on EPI-stimulated myocardial contractility in a time frame in which apoptosis could difficultly take place, thus making it difficult to compare ours with the results published by others. More generally, all these studies were performed in rodent *in vivo* models; therefore we have to be cautious in the eventual transfer of the results to the pathophysiology of human diseases. For instance, the experimental model of doxorubicin-induced acute cardiotoxicity chosen [107, 108] does not closely mimic the chronic cardiomyopathy observed in clinical situations during which repeated smaller doses of doxorubicin are given over a period of time, as reported by the same authors [100]. Finally, and most important, although it is well known that TPO primes the activity of other mediators on important biological effects in mature platelets and other cell types [30–32, 86], previously reported results were obtained in controlled experimental conditions, in which only the effects of TPO alone and at high doses were considered, while the combined effects of the administration of TPO with other soluble mediators were not evaluated [100–102]. On the contrary, we studied the effects of TPO contained in the plasma samples of patients with different pathological conditions [25, 64, 72, 85], and we were able to show the contribution of TPO to the biological effects studied by selectively inhibiting TPO activity with a TPOR-FC chimeric protein synthesized in our laboratory [25, 64, 72, 85]. Moreover, we investigated the effects of TPO on myocardial contractility at physiologic concentrations and in association either with an adrenergic stimulus (EPI) or the main cytokines known to depress myocardial activity in septic shock, that is, TNF-*α* and IL-1*β* [85].

**Figure 1:**
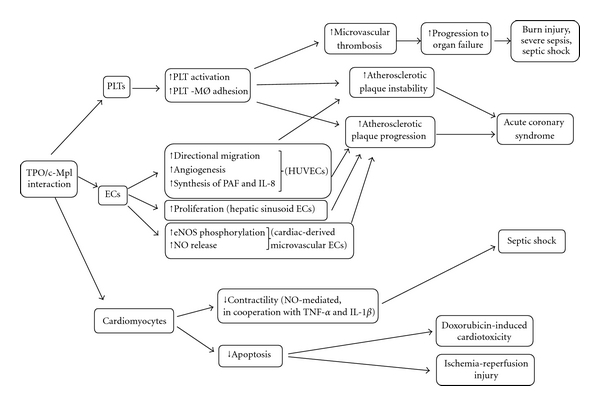
Schematic representation of TPO/c-Mpl functions in vascular system. PLTs = platelets; ECs = endothelial cells; MØ = monocytes; PAF = platelet-activating factor; IL-8 = interleukin-8; HUVECs = human umbilical vein-derived endothelial cells; NO = nitric oxide; TNF-*α* = tumor necrosis factor-*α*; IL-1*β* = interleukin-1*β*.

Therefore, our findings stress the importance of careful evaluation of the cardiovascular effects of TPO* in vivo*, especially in the clinical setting of diseases, such as heart failure and septic shock, whose pathogenesis is complex and involves the activation of a cascade of soluble mediators (Figure 1). Interestingly, large-scale clinical trials evaluating the effects of monoclonal antibodies against TNF-*α* in chronic heart failure patients gave rather disappointing outcomes [131, 132], suggesting that counterbalancing this cytokine alone may not be sufficient [133]. The individuation of TPO as an additional molecular target may provide clue for the development of new therapeutic interventions for the treatment of patients with sepsis-associated cardiac dysfunction and eventually chronic heart failure. Interestingly, several recent studies proposed also new experimental therapeutic approaches to various cardiac diseases based on the modulation of the inflammatory and prothrombotic states which are often associated with their development and progression [134–136]. Moreover, a careful and complete evaluation of the biological effects of TPO* in vivo*, in particular for what concerns cardiovascular consequences, needs to be envisaged.

## Figures and Tables

**Table 1:** List of publications describing biological functions of TPO/c-MPL system apart from hematopoietic mechanisms.

Mature platelets	[12, 25, 30–32, 64, 72, 109–118]
Polymorphonuclear leukocytes	[86, 119, 120]
Endothelial cells	[96–99, 121, 122]
Cardiac cells	[85, 100–102]
Brain cells	[70, 123–127]
Ovarian cells	[128]
Cancer cells	[129, 130]
